# Cell Line and Media Composition Influence the Production of Giant Plasma Membrane Vesicles

**DOI:** 10.1021/acsbiomaterials.3c01596

**Published:** 2024-02-20

**Authors:** William Doherty, Sarah Benson, Lisa Pepdjonovic, Abigail N. Koppes, Ryan A. Koppes

**Affiliations:** † Department of Chemical Engineering, Northeastern University, 360 Huntington Avenue, Boston, Massachusetts 02115, United States; ‡ Department of Biology, Northeastern University, 360 Huntington Avenue, Boston, Massachusetts 02115, United States; § Department of Bioengineering, Northeastern University, 360 Huntington Avenue, Boston, Massachusetts 02115, United States

**Keywords:** giant plasma membrane vesicles, biofabrication, formulations

## Abstract

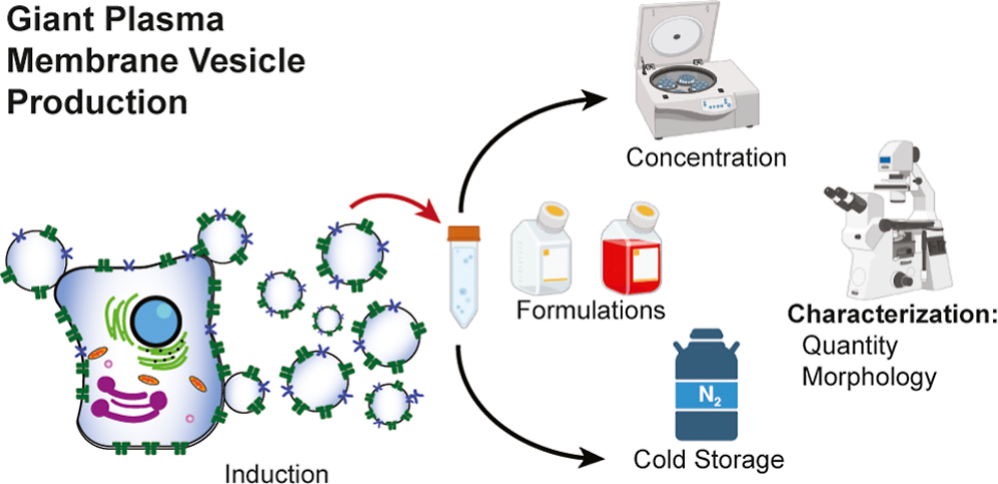

Giant plasma membrane vesicles (GPMVs) have been utilized as a model to study phase separation in the plasma membrane. Additionally, GPMVs have been employed as vehicle for delivering molecular cargo, including small molecule drugs and nanoparticles. Nearly all examples of GPMV production use a defined salt buffer that is a stark contrast to typical cell culture medium. In this study, we demonstrate that the addition of formaldehyde and dithiothreitol to a standard culture medium was capable of generating GPMVs at a concentration equal to or higher than the traditional production buffer. These methods were evaluated for two human cell lines: kidney endothelial and Schwann cells (SCs). Morphological properties of the resultant GPMVs exhibited no significant differences between the two formulations. Factors such as pH and seeding density significantly influenced the production of GPMVs in both mediums. The cell type and seeding density was shown to influence the number of GPMVs to the greatest extent. SCs yield more GPMVs at higher seeding densities compared to endothelial cells. Stability of the membrane of the GPMVs produced in both mediums was evaluated by monitoring passive diffusion of two fluorescently tagged dextrans (3 and 10 kDa). Regardless of the production formulation or cell type, approximately 85% GPMVs are impermeable to either dextran. Cold storage for on-demand use and shipping are essential for broader use of GPMVs. Toward this aim, we have evaluated the GMPV number and morphologies following storage at −80 °C and in liquid nitrogen. A significant loss of the GPMV number, ∼30%, was observed following storage across production formulations as well as cell types. Our results indicate that smaller GMPVs, <5 μm are more stable for preservation. In conclusion, GPMVs can be produced in a broad range of formulations, exhibit a high degree of stability, and can undergo cold storage for further adoption.

## Introduction

Giant plasma membrane vesicles (GPMVs) are cell-derived vesicles originally developed to isolate cell surface membrane fragments to study the plasma membrane of cells.^1−4^ While first produced almost 40 years ago, only within the last 15 years have GPMVs been heavily utilized as a plasma membrane model. What makes GPMVs unique and attractive is that they retain nearly all membrane components from the original cell they were derived from, including lipid composition, transmembrane proteins, and membrane channels.^5^ Additionally, GPMV membranes lack cytoskeletal entwinement, permitting the unrestricted migration of the membrane components.^1,6^ As a result, GPMVs provide a new way to study the composition and organization of the plasma membrane.^4^

GPMVs gained attention as a membrane model when Baumgart et al. observed a liquid–liquid phase separation into a cholesterol-rich lipid-ordered phase and a cholesterol-poor lipid-disordered phase in the GPMV membranes after they drop below a certain temperature.^1,7−10^ While it is hypothesized that these “raft domains” form spontaneously in cells, cytoskeletal entwinements limit their size, which is estimated to be on the nanoscale and thus is too small to detect. The GPMV membranes resemble those of the original cell membranes, a feature that has led to GPMVs being considered for other applications. They have shown potential as a tool for drug delivery, both as a vehicle and as a model to study the transport of drugs across the cell membrane.^11,12^ They have also been utilized to measure the mechanical properties of the cell membrane as GPMVs lack cytoskeletal entwinement that helps reinforce the membrane and thus interferes with taking these measurements.^13^ Finally, they have shown the potential to affect the local cellular environment, with GPMVs derived from INS-1 cells inhibited by the formation of islet amyloid polypeptides and osteoblast-derived GPMVs drive mesenchymal stem cells toward osteogenic differentiation.^14,15^

**Figure 1:**
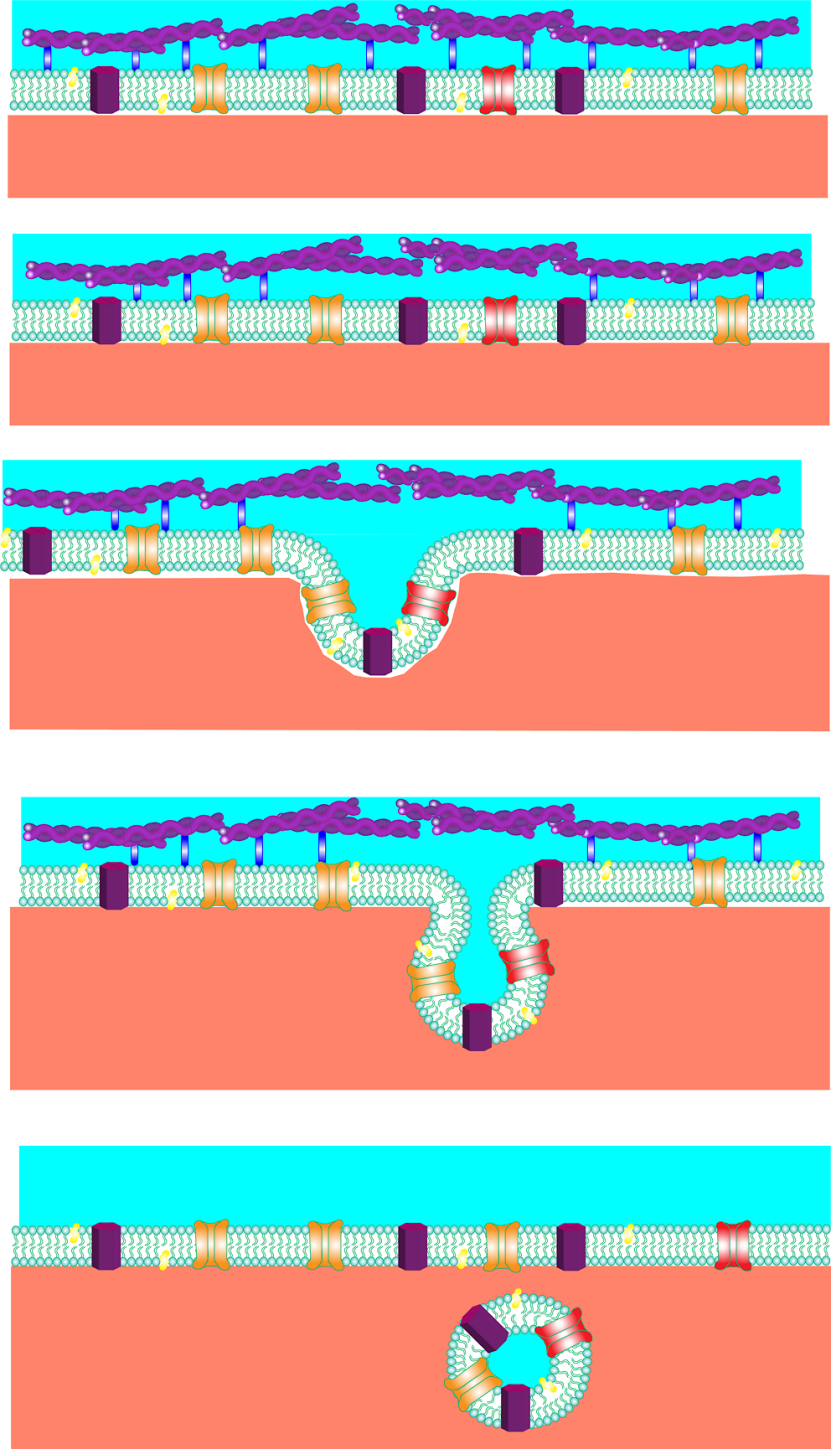
Overview of the hypothetical GPMV production mechanism. Localized detachments (blue bonds) from the cell cytoskeleton (purple) cause the membrane to bulge and pinch off from the cell. The GPMV membrane features components from the original cell including lipids (green), cholesterol (yellow), transmembrane proteins (dark purple), and membrane channels (red and orange).

GPMVs are produced by incubating cells with low concentrations of formaldehyde (PFA) and dithiothreitol (DTT).^1,16^ This is proposed to create a breakdown of the cytoskeletal entwinement in the plasma membrane and create small areas of structural weakness (Figure 1).^13^ The intercellular pressure then pushes on the weakened areas and the membrane begins to bulge.^15,17^ In time, the membrane will pinch off from the cell, encapsulating the cytoplasm of the cell within that piece of membrane to form vesicles ranging from 3 to 20 μm in diameter.^18,19^ While the number of potential applications for GPMVs has blossomed over the years, their primary focus is still on the study of the inner workings of the plasma membrane with few applications outside of that perspective. We believe there are potential applications for GPMVs as tools used with living cells that have not been given much consideration. This may be in part to the buffer used to generate them being unable to support in vitro cell culture for long periods of time as it lacks glucose and serum among other important components used to culture cells.^20,21^ We also question whether the defined salt buffer is the most advantageous base medium for GPMV production. While studies up to now may not require a large number of GPMVs, future studies could benefit from alternative GPMV production strategies that produce GPMVs in greater numbers and/or GPMVs that are more stable in cell culture medium, with an example being GPMVs used as drug delivery vehicles.^22^

In this study, we wanted to explore the feasibility of producing GPMVs in DMEM-based cell culture medium by adding PFA and DTT to the culture medium. We compared GPMV production in culture media directly with those produced using the traditional salt buffer. We studied how incubation time, pH, and cell density influenced the number of GPMVs generated from human embryonic kidney 293T cells (HEK293T) and human neurofibromatosis 1 cells (hNF1), as well as if there were any changes in their size and circular properties. We chose these two cell lines for their contrasting cell morphologies, contact affinity, and motilities to compare the number of GPMVs produced and their physical characteristics after GPMV formation. Additionally, HEK293T cells are easily transduced, enabling the expression of foreign membrane proteins which could be useful in studies on membrane components.^23−25^ Additionally, we determined how both production methods affected GPMV permeability; the effect of centrifugation speeds on the number of GPMVs recovered in resuspension; and the stability of GPMVs when they were undergoing cryogenic freezing and subsequent thawing. These studies aim to expand on how GPMVs can be produced, highlight parameters that change GPMV production, and show how different production methods can affect the physical properties of GPMVs.

## Methods

### Cell Culture

HEK293T cells (ATCC) and human NF1 Schwann cells (SCs) (ATCC) were cultured in a growth medium (GM) composed of DMEM supplemented with 10% fetal bovine serum (FBS) and 1% penicillin–streptomycin. Media exchanges were performed every 2–3 days, and cells were passaged at 90% confluency.

### GPMV Production

GPMVs were produced by using two formulations. The first was a buffer comprised of 10 mM HEPES, 2 mM CaCl_2_, 150 mM NaCl, 25 mM PFA, and 2 mM DTT. When producing GPMVs with this buffer, cells were washed twice in a washing buffer (10 mM HEPES, 2 mM CaCl_2_, and 150 mM NaCl) before adding the production buffer (PB) to the cells and then incubated at 37 °C and 5% CO_2_. The second formulation added 25 mM PFA and 2 mM DTT to the medium used for cell culture (DMEM, 10% FBS, and 1% P/S). When using this formulation for GPMV production, the standard cell culture medium was removed, and the production medium (PM) was added directly to the cells and incubated at 37 °C and 5% CO_2_. Unless otherwise stated, the cells were incubated for 4 h in PB and PM. The PM was removed, and the suspension was centrifuged at 500*g* for 5 min to pellet any large pieces of cell debris that detached from the flask. The buffer was then removed without disturbing the pellet and aliquoted into 1.5 mL centrifugal tubes. The tubes were spun down at 17,000*g* for 30 min at 4 °C. The buffer was aspirated off, and the pellet was resuspended in 200 μL of washing buffer or culture medium for later use.

### GPMV Imaging and Morphological Analysis

For each trial, a 10 μL of sample was placed on thin glass cover slides and covered with an 18 mm coverslip. The sample was placed on the stage and allowed to settle for several minutes. Brightfield images of the slide were taken using a Zeiss Microscope at 20× magnification. Each image was analyzed with MATLAB’s image analysis toolbox using the regionprops function to determine the number and physical properties of the GPMVs.

### GPMV Production over Time

HEK293T and hNF1 cells were grown to roughly 70–80% confluency on a 6-well tissue culture plate. On the day of GPMV production, cells were incubated with 1 mL of PB or PM and then placed in an incubator at 37 °C and 5% CO_2_ for the allotted time. Every 2 h, one well of suspension was removed starting from hour 2 until hour 10. The samples at hour 2 and hour 4 were kept at 4 °C and then after the hour 6 sample was taken, they were all processed simultaneously. Samples were spun down and resuspended in 200 μL of washing buffer for the PB/PB samples or growth media for the PB/PM and PM/PM samples. After resuspension, eight images of each sample were taken and analyzed to determine GPMV concentration and measure the physical characteristics of the GPMVs.

### Repeated GPMV Production

HEK293T and hNF1 cells were grown to 70–80% confluency on a 6-well tissue culture plate. Fresh PB and PM were made and aliquoted into 1 mL volumes and kept at 4 °C until needed. Every 2 h, the buffer was collected, and fresh PB or PM was added to the cell monolayer. Samples were analyzed as described above. This was repeated up to five times (10 h of incubation). Cell monolayers were monitored at each exchange for observable changes in cell density; however, no measurable changes were seen. For production from HEK293T, no GPMVs were observed after hour 6.

### Influence of pH on Production

HEK293T cells were plated in a 6-well plate and grown to 70–80% confluency. For all studies, PB and PM were made fresh on the day of the experiment and were pH adjusted using 1 M NaOH. For each well, 1 mL of PM was placed on the cells, and each well was incubated for 2 h at 37 °C and 5% CO_2_. The GPMVs were collected, pelleted, and resuspended in 200 μL of buffer/medium at the pH they were produced at. Samples were taken from each sample and eight brightfield images of each sample were taken and analyzed by MATLAB to determine the GPMV concentration.

### Cell Density’s Influence on GPMV Production

HEK293T and hNF1 cells were seeded and grown to confluency. The cells were passaged with trypsin–ethylenediaminetetraacetic acid and reseeded at 10% of the cell volume. For the following 4 days, one sample for each cell type was lifted and counted to determine the cell density of the samples. Samples were incubated in GPMV PM for 4 h before being processed for sampling. Each condition had three samples that were imaged with brightfield and then analyzed using MATLAB to determine the concentration and morphological properties of the GPMVs collected.

### Seeding Density

HEK293T and hNF1 cells were grown to confluency. The cells were lifted, pooled, and then reseeded on 6-well plates with a seeding density of 1.2 × 10^5^, 3 × 10^5^, 6 × 10^5^, 9 × 10^5^, and 10.8 × 10^5^ cells for the individual wells on the plate. The cells were incubated overnight before being taken for GPMV production the following morning. Two well plates produced GPMVs in PB, and the third used PM, and all three well plates were incubated for 4 h before production. The samples were removed from the well plate, spun down, and then resuspended in the desired buffer. For each condition, three samples were used, and brightfield images were taken of each sample and then analyzed using MATLAB to determine the concentration and characteristic properties of the GPMVs collected.

### GPMV Permeability

HEK293T and hNF1 cells were grown to 70–80% confluency in T-25 culture flasks before being incubated in PB and PM for GPMV production. GPMVs were collected and aliquoted into three1.7 mL centrifugation tubes. Fluorescein-labeled dextran (3 kDa)and Alexa Fluor 647-labeled dextran (10 kDa) molecules were added to each suspension at a final concentration of 25 μL/mL (Figure S1). After addition of dextran, one tube was centrifuged at 17000*g* for 30 min at 4 °C and then resuspended in washing buffer for GPMVs produced in PB and in complete cell culture medium for GPMVs produced in PM. The other two samples remained suspended in the dextran solution for 1 and 2 h, respectively, before being spun down and resuspended. The GPMVs were imaged as mentioned previously with fluorescent images for fluorescein and Alexa Fluor 647 in addition to the standard brightfield images to determine the number of GPMVs containing dextran.

### Influence of Centrifugation on Yield

GPMVs were produced from HEK293T cells in 6-well plates using PB and PM. GPMVs were collected and aliquoted into a 1.7 mL centrifugation tube. A 50 μL of sample was taken from each tube to measure the initial GPMV concentration. The rest of the suspension was centrifuged at the desired centrifugation speed for the trial for 5 min. The production solution was aspirated off and placed in a second centrifugation tube, and the pelleted GPMVs were resuspended in 190 μL of washing buffer and culture media for PB and PM GPMVs, respectively. Two 10 μL of samples of each sample were prepared, and 10 brightfield images of each sample were taken for analysis. When making comparisons between samples, the GPMV concentration was reduced to a fifth of the measured concentration to represent the 1× dilution at which the other two samples were imaged at.

### Cryogenic Storage

For cryopreservation, GPMV stability was tested when stored at −80 °C and in liquid nitrogen (LN) as well as the effect of the addition of the cryopreservation agent dimethyl sulfoxide (DMSO) (10% v/v) and a controlled-rate freezing container (Mr. Frosty freezing container with isopropyl alcohol). The total GPMVs generated were split into four groups: one was frozen without either the cryopreservation agent or the freezing container, one with DMSO, one in the freezing container, and the last with DMSO and using the freezing container. The samples were frozen and left at −80 °C and in LN for 3 days before they were thawed on ice and imaged.

### Sample Populations and Statistical Analysis

For each analysis, three full technical replicates were completed (*n* = 3), and eight images were taken for each sample (*m* = 24). All statistical analysis was performed in PRISM, comparing the means for each of the three trials. Data were tested for normality, and differences were identified with a one-way analysis of variance (ANOVA) and Tukey’s comparisons of means test.

## Results

### GPMV Production over Time

**Figure 2:**
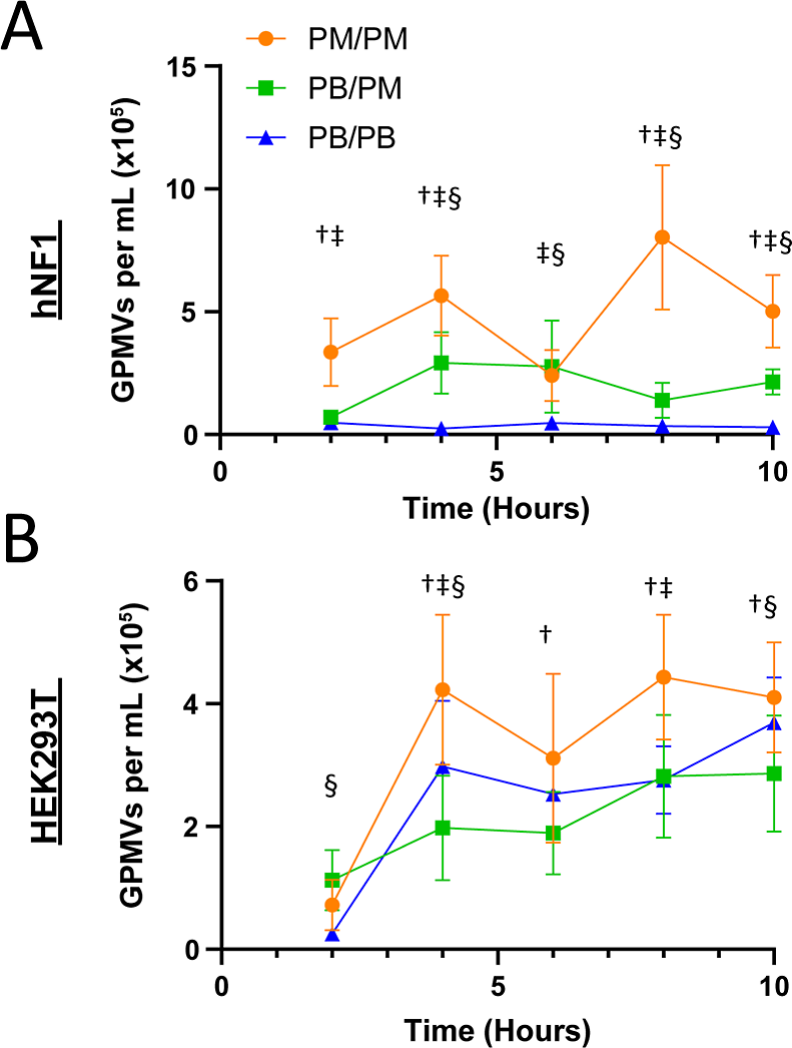
GPMV concentration in (A) hNF1 and (B) HEK293T cells show no significant increase after 4 h of incubation in both PMs. Samples after 6, 8, and 10 h of incubation remained consistent with samples taken at hour 4 in both cell types across the three production conditions (*n* = 3, *m* = 24; mean ± S.D; one-way ANOVA, *p* < 0.05 across formulations: †—PM/PM vs PB/PM; ‡—PM/PM vs PB/PB; §—PB/PM vs PB/PB).

GPMV production, specifically the overall concentration, is influenced by both the cell type and induction media (Figure 2). GPMVs were produced in PB, PM, and in PB while resuspending them in PM to study how GPMV production changes with incubation time in the PM. After 4 h of inducing GPMVs, the number of GPMVs produced from both cell types plateaued with only minor fluctuations observed at longer incubation times greater than 4 h (Figure 2A). GPMVs produced using PB did not have a significant increase in concentration after 4 h of incubation in the buffer for either cell type (Figure 2A,B). On average, an increase in GPMV concentration was observed for production in PM versus PB for both the PB/PM and the PB/PB conditions. In GPMVs produced from HEK293T cells, the PB/PM condition had the lowest concentration from hours four to ten, hinting that GPMVs produced from PB may have stability issues when resuspended in a different medium.

**Figure 3:**
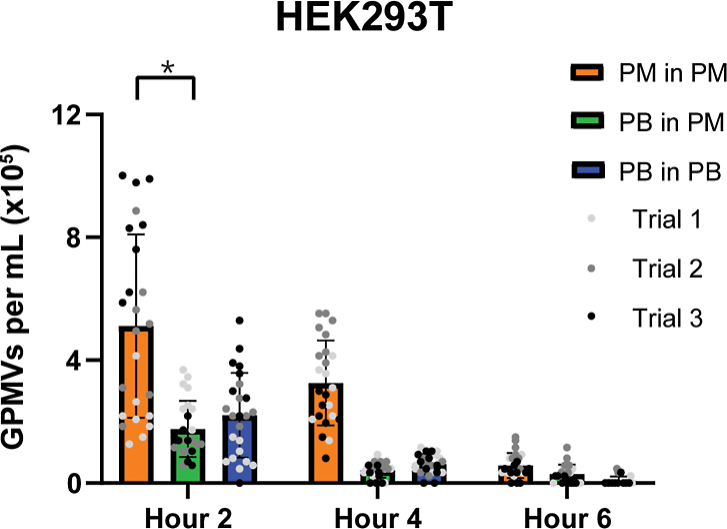
Effect of exchanging PM over time on GPMV production: GPMVs from HEK293T cells were generated with the PM fully exchanged every 2 h. After 4 h of incubation (two full media exchanges), a significant reduction in GPMV production is observed in both mediums (*n* = 3, *m* = 24; *=*p* < 0.05, **=*p* < 0.01, ****=*p* < 0.001).

With the rate of GPMV production being stunted after 4 h, we hypothesized that this could be due to the consumption of the vesiculation agents of PFA and DTT during the blebbing process. To test this, HEK293T cells were induced to form GPMVs using the same formulations; however, a full media exchange was carried out at each 2 h time point. Similar to partial sampling of vesiculation, a full exchange of the media yielded higher concentrations in the first 4 h of incubation (Figure 3). An approximate 2-fold increase in GPMV concentrations was observed in the PM formulation compared to PB/PM and PB/PB. Following the exchange at hour four, significantly fewer GPMVs were collected at hour six and no GPMVs after hour eight.

### GPMV Production, Size, and Circularity between PB and PM

A direct comparison between the two formulations showed that GPMVs produced from HEK293T cells were higher in concentration when using the cell culture medium for production compared to the defined salt buffer commonly used for GPMV production. GPMVs produced from and resuspended in DMEM averaged 7.48 ± 1.21 × 10^5^ GPMV per mL while GPMVs produced using a PB averaged 5.72 ± 1.36 × 10^5^ and 5.82 ± 1.22 × 10^5^ GPMVs per mL under the PB/PB and PB/PM conditions, respectively. With hNF1 cells, GPMV concentration decreased for PB/PB & PM/PM conditions. GPMVs produced and resuspended in media averaged 1.07 ± 0.428 × 10^5^ GPMVs per mL. GPMVs produced using the PB averaged 51.1 ± 2.06 × 10^4^ GPMVs per mL when resuspended in media and 4.22 ± 1.68 × 10^4^ GPMVs per mL when resuspended in PB. During the refeeding studies, we saw the opposite trend with the GPMV concentration from hNF1 cells produced in PB being more than double the concentration of GPMVs produced in PM. While the number of GPMVs varied greatly from cell to cell, there was a higher yield of GPMVs from HEK293T cells when using basic media compared to the PB, while GPMVs from hNF1 cells showed comparable but inconsistent yields using both methods.

**Figure 4:**
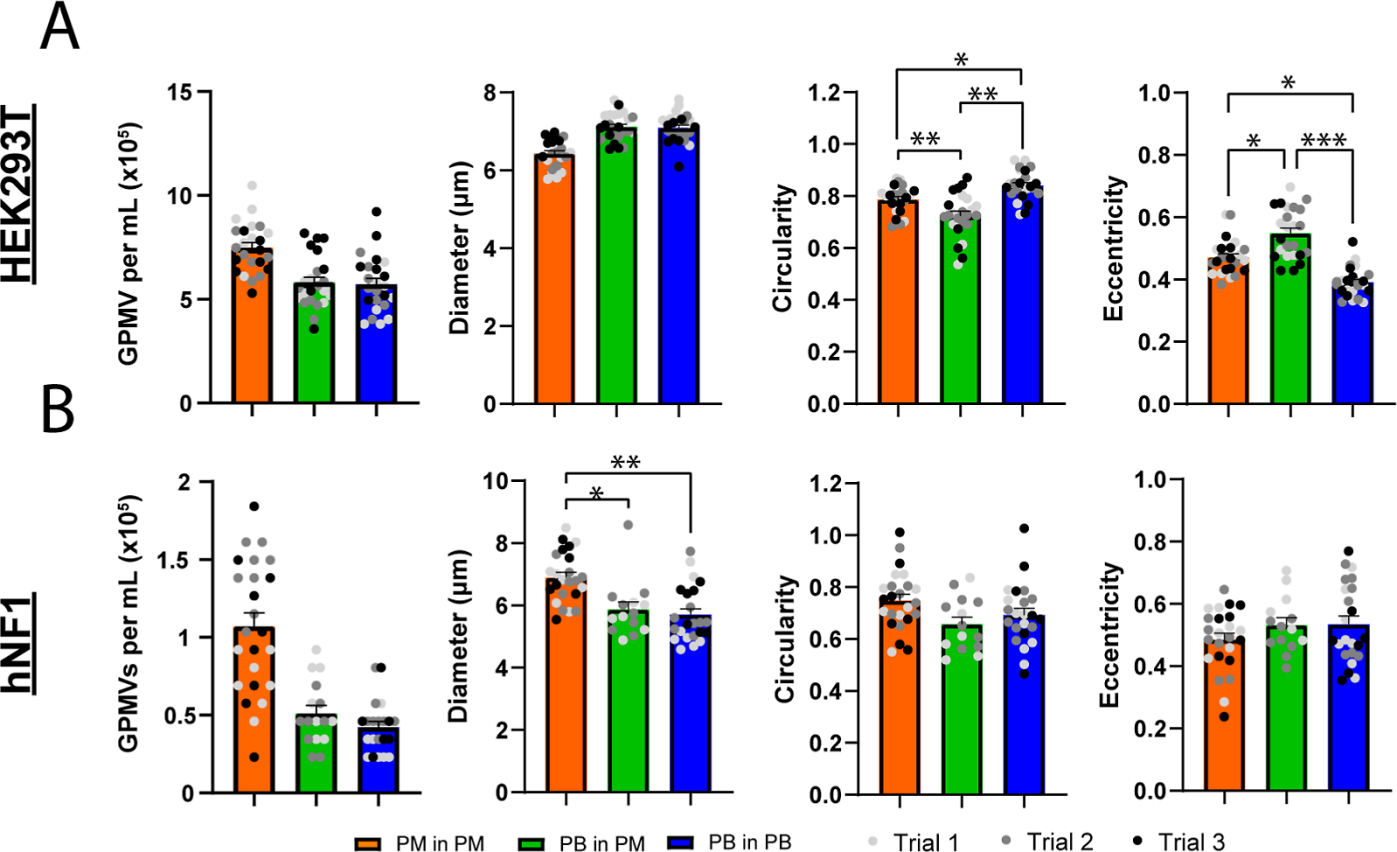
GPMV properties produced in PB and PM from HEK293T (A) and hNF1 (B) cell lines. GPMV concentrations were analyzed after 4 h of incubation, and the average diameters, circularity, and eccentricity were measured. Image analysis was done using MATLAB, and statistical analysis was done using one-way ANOVA (*n* = 3, *m* = 24; *=*p* < 0.05, **=*p* < 0.01, ****=*p* < 0.001).

As an indication of the GPMV stability, the morphological properties of the GPMVs produced using PM and PB were found with the MATLAB Image Analysis Toolbox. GPMVs were generated from both cell lines, and the GPMV concentration for each production condition are shown for HEK293T cells (Figure 4A) and in hNF1 cells (Figure 4B). In the HEK293T cells, the GPMVs had an average diameter of 6.2 ± 0.22 μm when produced and resuspended in basic media, while the GPMVs produced in the PB averaged a diameter of 6.7 ± 0.22 μm (Figure 4). While the average diameter decreased when produced in PM, the number of GPMVs produced increased. GPMVs from hNF1 cells show the opposite trend with the average diameters of GPMVs formed in PM around 6.4 ± 0.04 μm while GPMVs from the PB averaged diameters of 5.9 ± 0.4 μm (Figure 4). From both cell types, the diameter of GPMVs produced using PM were similar despite differences in the size and morphology of HEK293T and hNF1 cells. When produced in PB, we saw a noticeable difference in GPMV diameter with GPMVs from hNF1 cells being 0.8 μm smaller on average than GPMVs produced from HEK293T cells. GPMVs from each cell line and formulation were further characterized and evaluated for their circularity and eccentricity (Figure 4). GPMVs produced from HEK23T cells in the PB/PB formulation exhibit a higher degree of roundness (higher circularity and lower eccentricity) compared to other PM/PM and PM/PB. However, these significant differences are most likely driven by high sample numbers (*m* = 24). No differences are observed in GPMVs produced from hNF1 cells across the different formulations.

### pH’s Influence on GPMV Production

**Figure 5:**
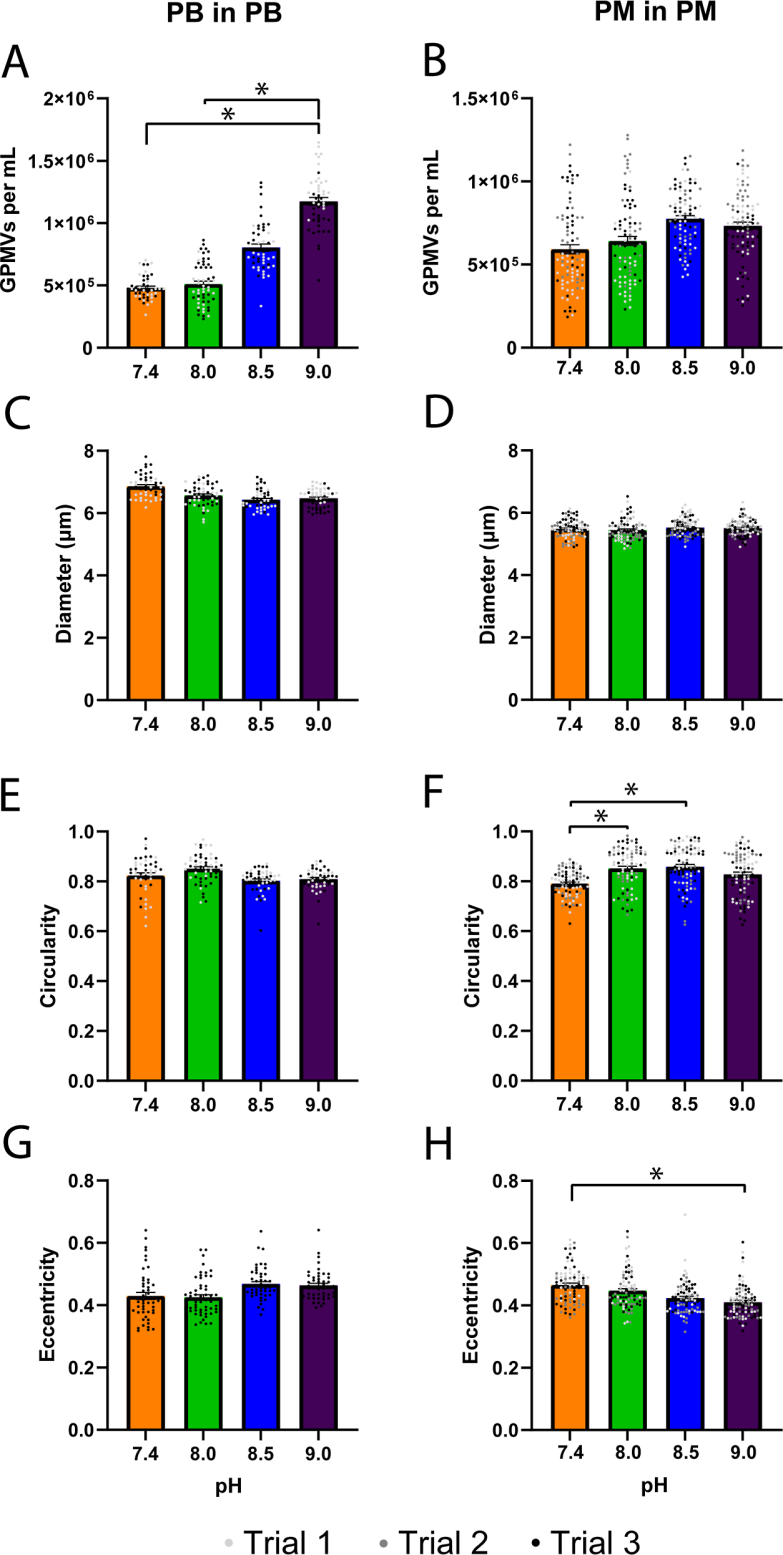
pH effect on GPMV production. GPMVs produced at pH 7.4, 8.0, 8.5, and 9.0 in (A) PB and (B) PM. The GPMV (C,D) diameters, (E,F) circularity, and (G,H) eccentricities are shown for GPMVs produced at pH 7.4, 8.0, 8.5, and 9.0 in PB and PM (*n* = 3, *m* = 24; *=*p* < 0.05, **=*p* < 0.01, ****=*p* < 0.001).

Increases in the pH have been shown to affect GPMV production. Production in PB increases with increasing pH, while raising the pH of a full cell medium formulation exhibits no change on the number of GPMVs produced (Figure 5). A 2-fold increase in GPMVs generated was observed when increasing the pH from 7.4 to 10 in PB (Figure 5A). When produced in PM of varying pH, we saw an increase in the GPMV concentration between 8 and 8.5 (Figure 5B). While there was a noticeable increase in the concentration of GPMVs, there was no noticeable change in any of the physical characteristics of the GPMVs associated with a change in pH.

The average GPMV diameter was approximately 6.2 μm across all four pH values using PB (Figure 5C) and 5.8 μm in GPMVs produced in PM (Figure 5D). While there were some changes in diameter, circularity, and eccentricity using both mediums, there was no noticeable trend that could be correlated with the increased pH outside of the concentration of GPMVs.

### Cell Confluency and GPMV Production

**Figure 6:**
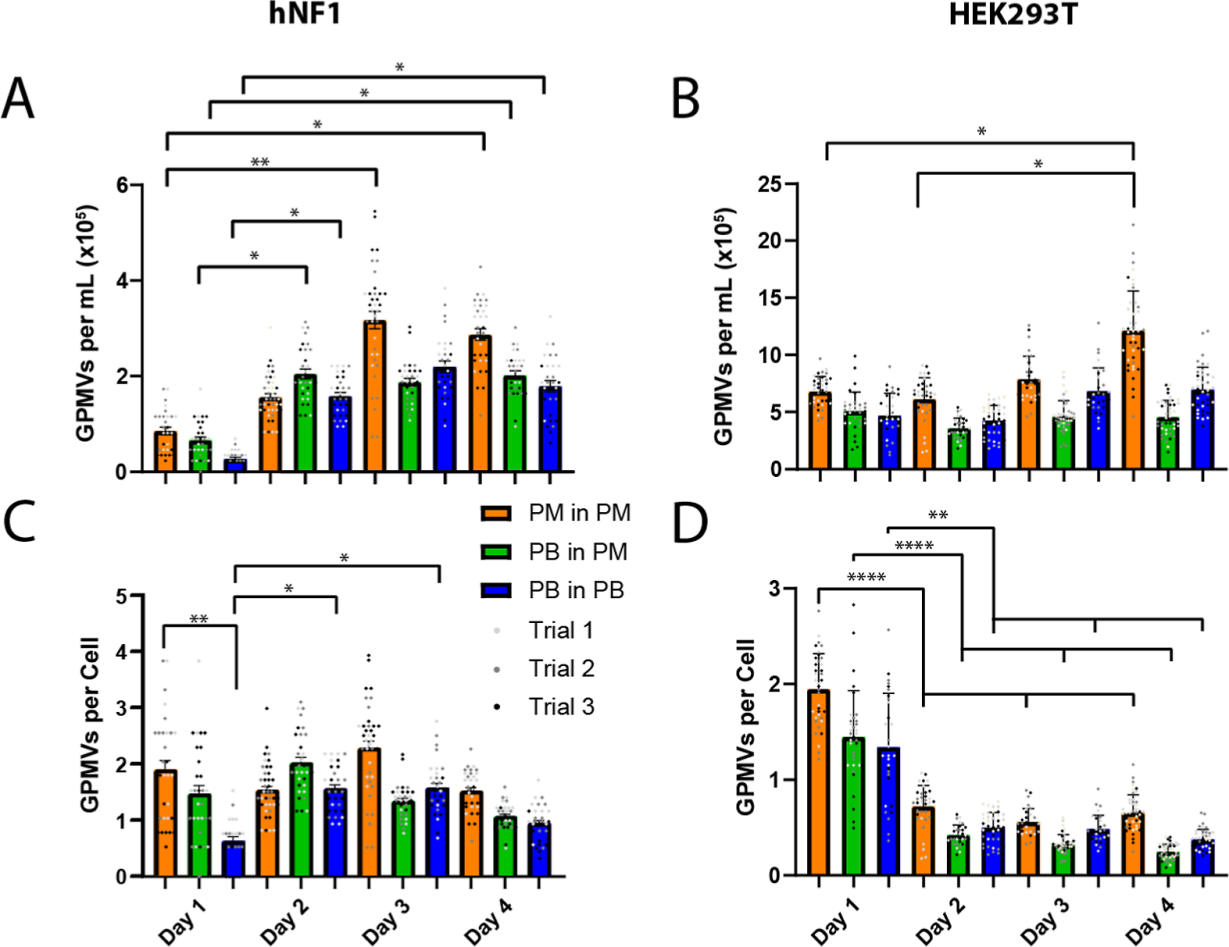
GPMV production at varying days after seeding. GPMVs were produced from (A) hNF1 and (B) HEK293T cells seeded in 6-well plates at 10% confluency. Each day a cell count was taken and used to determine the number of GPMVs produced per cell of (C) hNF1 and (D) HEK293T cells. (*n* = 3, *m* = 24; * = *p* <.05, ** = *p* <.01, **** = *p* <.001).

As the cells grew to confluency, an increase in the concentration of GPMVs harvested was observed from both cell types (Figure 6A,B). However, when the total GPMV were normalized to a per-cell basis, a significant reduction was present in GPMV yield for HEK293T cells as cells grew to confluency (Figure 6C,D). While producing GPMVs at higher confluency still resulted in a higher yield of GPMVs, the number of GPMVs per cell decreased as the cells grew to confluency. Unlike the GPMVs produced from the HEK293T cells, the number of GPMVs produced per hNF1 cell stayed consistent regardless of cell density.

**Figure 7:**
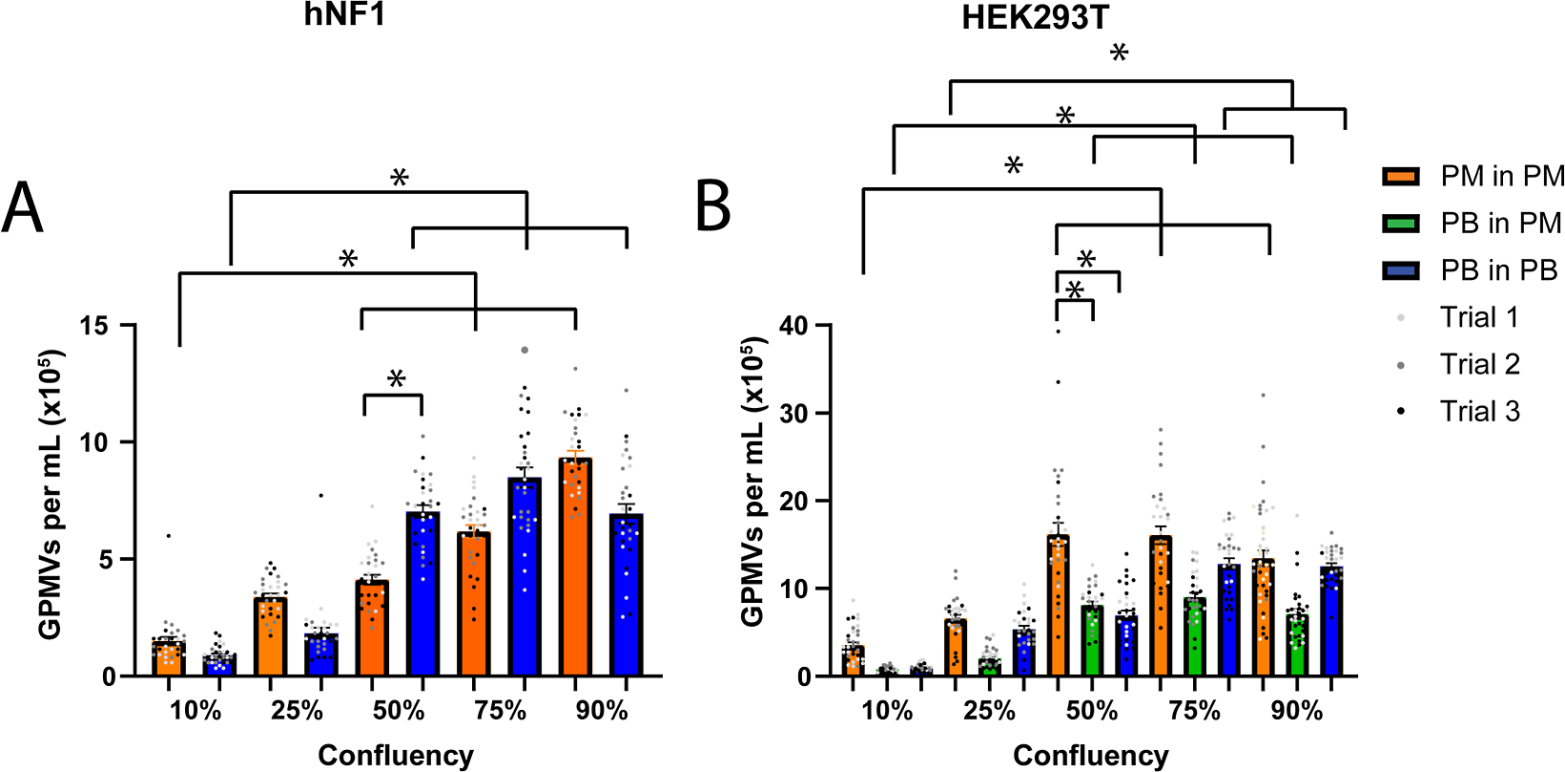
GPMV production increases with increasing cell densities. GPMVs were produced from (A) hNF1 and (B) HEK293T cells seeded in 6-well plates at fractions based on the number of cells when the cells reach confluency. A large increase in GPMV production for PM over PB is observed in HEK293T cells at 50%. Each well was sampled three times (*n* = 3, *m* = 24; mean ± S.D; one-way ANOVA; *=*p* < 0.05, **=*p* < 0.01, ****=*p* < 0.001).

The higher the seeding density, the more GPMVs were generated; however, this was not a linear increase (Figure 7A,B). Unlike with the previous study, we did reach a peak in GPMV production from the HEK293T cells seeded between 6 × 10^5^ and 9 × 10^5^ cells per well with the GPMV concentration decreasing when seeding at 1.1 × 10^6^ cells per well (>90% confluency). With the hNF1 cells, the total number of GPMVs increased with cell density and did not reach the same peak with the increased cell seeding density.

### GPMV Permeability

**Figure 8:**
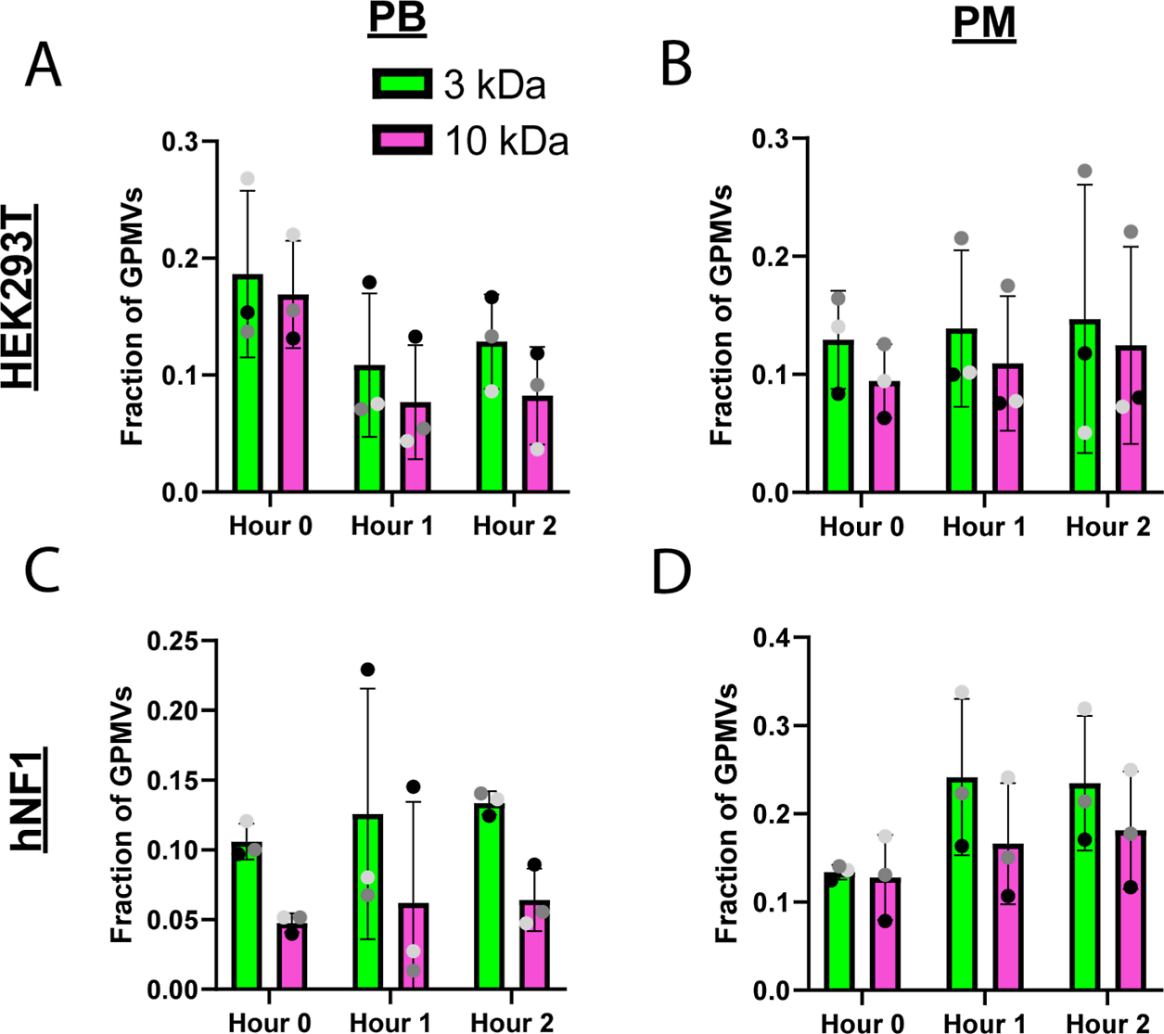
GPMV permeability to 3 and 10 kDa fluorescent dextran remains consistent across production formulations. GPMV permeability when suspended with 3 kDa fluorescein-tagged and 10 kDa Alexa Fluor 647-tagged dextran produced from HEK293T cells in PB (A) and PM (B) and from hNF1 cells in PB (C) and PM (D). Permeability was measured in GPMVs immediately after suspension in dextran mixture; after 1 h in dextran mixture; and after 2 h in dextran mixture.

GPMVs from both HEK293T and hNF1 cells were incubated with 3 and 10 kDa dextrans that were fluorescently tagged with fluorescein and Alexa Fluor 647, respectively. To ensure passive diffusion was not limited, three time points were studied (up to 2 h of incubation). Initially, 18.6 ± 7.1% of GPMVs produced from PB were permeable to 3 kDa dextran and 16.9 ± 4.6% permeable to 10 kDa dextran (Figure 8A). We observed 12.9 ± 4.1 and 9.4 ± 3.1% of GPMVs produced in PM were permeable to the 3 and 10 kDa dextran, respectively (Figure 8B). GPMVs produced from hNF1 cells using PB did not show a noticeable change in their permeability with 10.6 ± 1.3% initially, 12.6 ± 9% after 1 h, and 13.4 ± 0.8% after 2 h were permeable to 3 kDa dextran and 4.8 ± 0.7, 6.2 ± 7.2, and 6.4 ± 2.2% to 10 kDa dextran for each of the time points (Figure 8C). Regardless of the experimental condition and duration of incubation with fluorescently tagged dextrans of two different sizes, up to 20% of GPMVs are highly porous.

### Centrifugation of HEK293T GPMVs

**Figure 9:**
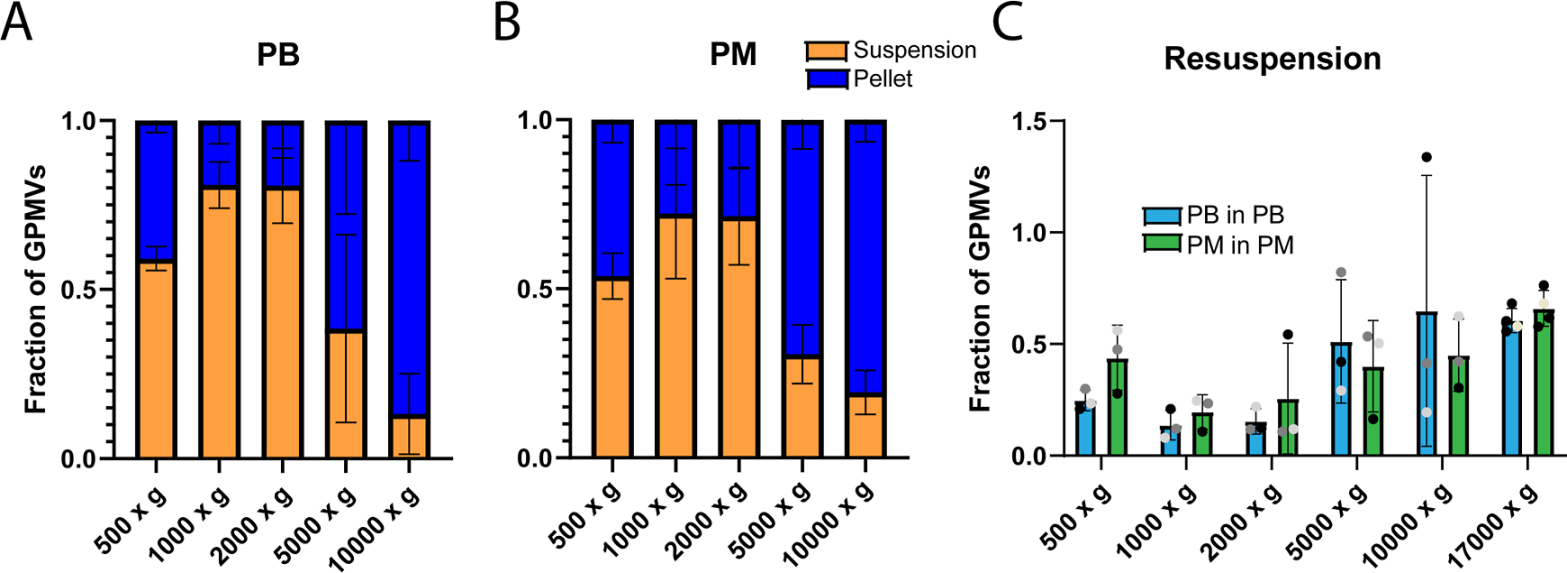
Centrifugation speed effect on GPMV yield: GPMVs produced in PB and PM were spun at various centrifugation speeds for 5 min. We see the fraction of GPMVs that were pelleted and resuspended vs the GPMVs that remained in the aspirate for GPMVs produced in PB (A) and PM (B). The average GPMV concentration in the resuspension was normalized to the initial GPMV concentration to determine the fraction of GPMVs lost during pelleting (C).

GPMVs were centrifuged at different speeds to determine what fraction of GPMVs were captured in the pellet versus those remaining in the media. A higher fraction of GPMVs produced in PB and PM spun down at 500*g*, 1000*g*, and 2000*g* remained in suspension with a small concentration of GPMVs being pelleted for resuspension (Figure 9A,B). Once the centrifugation speed exceeded 5000*g*, a larger concentration of GPMVs were captured in the pellet. GPMVs produced in PB when centrifuged at 5000*g* resulted in 1.5 times the number of GPMVs in the resuspension compared to the aspirate and spinning at 10,000*g* resulted in ∼85% of the combined total GPMVs present in the resuspension. GPMVs produced in PM when spun down at 5000*g* had 70% of the total GPMVs present in the resuspension and 80% when spun at 10,000*g*. When spun at 17,000*g*, we observed no GPMVs in the aspirate using either medium, and thus we did not show this data in Figure 9A,B. When comparing the concentration in the resuspension vs the initial concentration, there was a higher fraction of the initial number of GPMVs in the resuspension compared to the fraction recovered when produced in PB when spun down at 500*g* and 1000*g* (Figure 9C). When the centrifugation speed was increased to 2000*g*, 5000*g*, and 10,000*g*, there was a higher fraction of GPMVs produced in PB recovered after resuspension, with the largest difference being for the 5000*g* spin where the fraction of PB GPMVs recovered was 0.47, while for PM GPMVs it was only 0.33. Finally, for both conditions, the highest fraction of GPMVs recovered at any of the centrifugation speeds was when spinning at 17000*g*, where 62% of PB GPMVs were recovered after resuspension and 68% recovered for GPMVs produced in PM. While there was no centrifugation speed examined that did not have a significant reduction in the fraction of GPMVs recovered, 17000*g* was the best speed for recovering the highest fraction of GPMVs despite concerns that spinning at such high speeds would potentially damage or destroy the GPMVs we are testing.

### Cryogenic Storage of GPMVs

**Figure 10:**
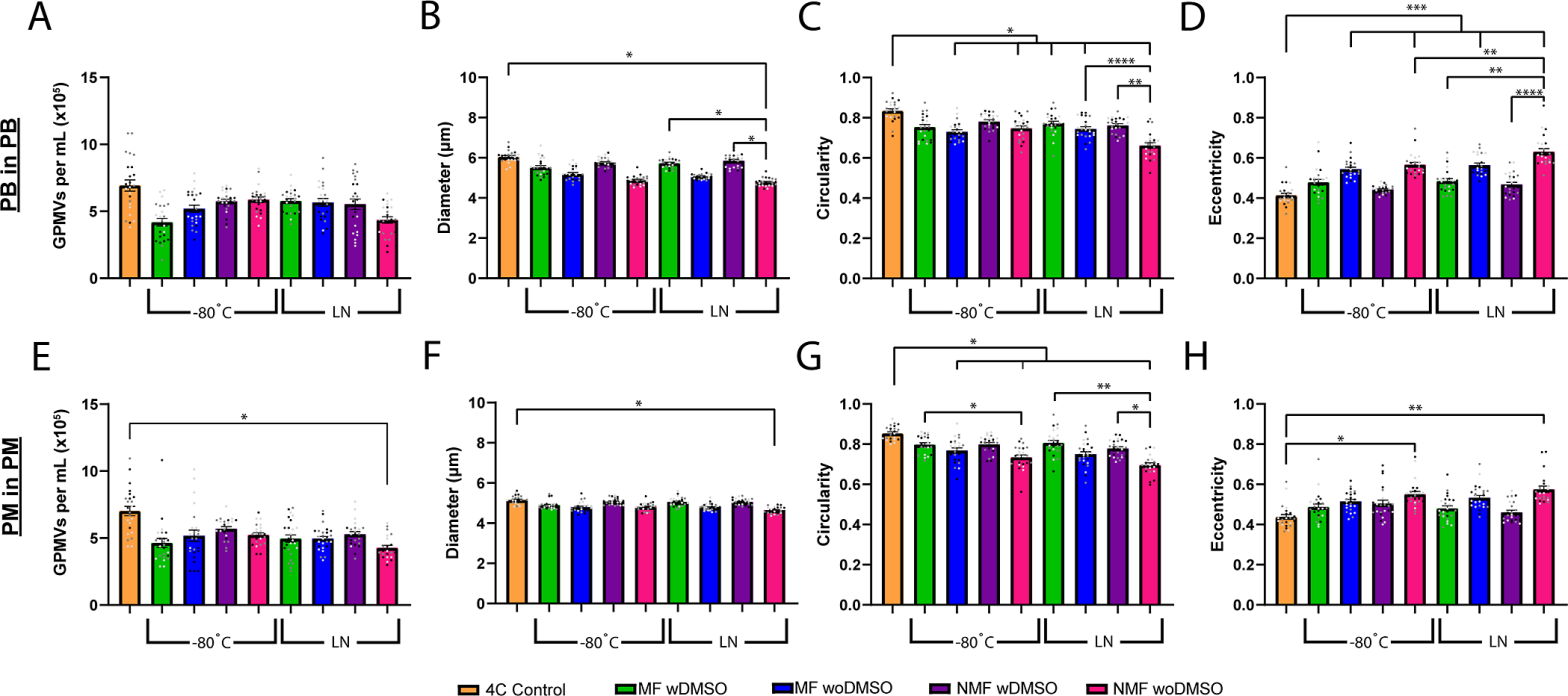
Cryogenic storage of GPMVs from HEK293T cells. GPMVs were generated in PB (A–D) and PM (E–H) and stored at −80 °C and in LN. The GPMV concentration (A,E), diameter (B,F), circularity (C,G), and eccentricity (D,H), were measured from samples on the day of generation and after 3 days of cryogenic storage. Image analysis performed in MATLAB and PRISM was used for statistical analysis.

The stability of GPMVs produced from HEK293T was further tested by performing cryogenic freezing and thawing at −80 °C and in LN as well as determining the feasibility of storing GPMVs in a cryogenic environment for an extended time. Under both cryopreservation conditions, a decrease in average GPMV concentration was seen from the initial concentration on day zero; however, GPMVs stored in LN show a larger decrease in average concentration compared to those stored at −80 °C (Figure 10A,E). GPMVs stored at −80 °C decreased in concentration by roughly 20% when produced in either PB or basic media and that increased to a 30% loss in concentration when stored in LN. There was a decrease in GPMV diameter of up to 10% in PM and 20% in PB (Figure 10B,F), while the circularity (Figure 10C,G) and eccentricity (Figure 10D,H) were also affected with the circularity decreasing as much as 20% in PM and 23% in PB, while the eccentricity increased over 30 and 50% in PM and PB, respectively.

**Figure 11:**
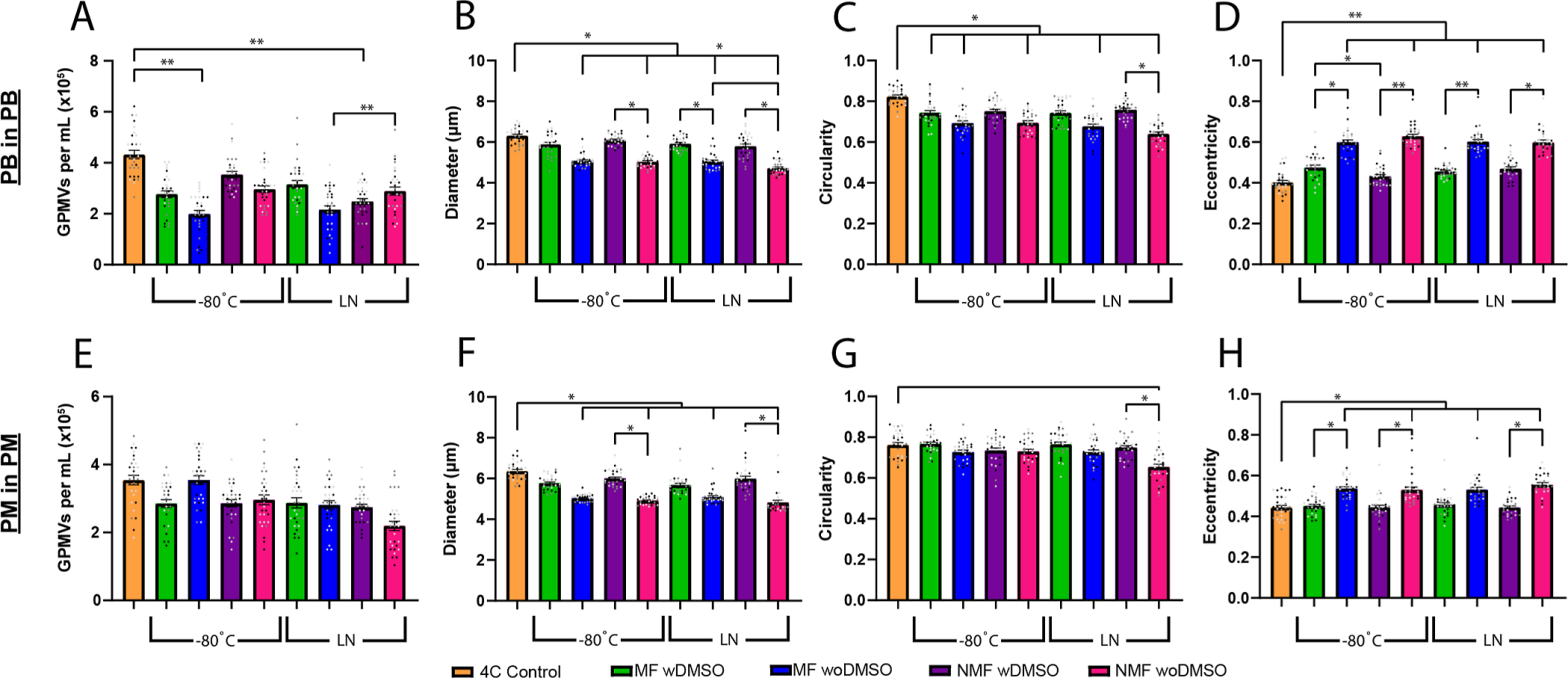
Cryogenic storage of GPMVs from hNF1 cells. GPMVs were generated in PB (A–D) and PM (E–H) and stored at −80 °C and in LN. The GPMV concentration (A,E), diameter (B,F), circularity (C,G), and eccentricity (D,H) were measured from samples on the day of generation and after 3 days of cryogenic storage. Image analysis performed in MATLAB and PRISM was used for statistical analysis.

GPMVs produced from hNF1 cells also underwent cryogenic freezing for 3 days at −80 °C and in LN. The GPMVs produced from PB and PM saw a significant reduction in GPMV concentration from day 0 to day 3 at −80 °C. Unlike with HEK293T cells, GPMV produced in PB did not further decrease in concentration when stored in LN; however, GPMVs produced in PM still show an additional decrease in concentration between −80 °C and LN (Figure 11A,E). Like with the HEK293T GPMVs, the hNF1 GPMVs experienced a decrease in GPMV diameter as much as 25% in both PB and PM (Figure 11B,F). This extended to the circular properties of the GPMVs as the circularity decreased over 15% in PM and 20% in PB (Figure 11C,G), and the eccentricity increased 25% and over 50% after freezing in both PM and PB, respectively (Figure 11D,H).

The effects of using the cryopreservation agent DMSO and using a polycarbonate freezing container filled with isopropyl alcohol to control the rate of freezing of GPMVs were also investigated. The concentration of GPMVs from HEK293T cells and hNF1 cells using PM that survived the freeze/thaw process was generally unaffected by either cryopreservation agent. For the GPMVs formed from hNF1 cells in PB, the concentration was higher between trials with and without DMSO at −80 °C and in LN, while a freezing container seemed to have no impact; however, GPMVs produced in PM did not show a similar trend for the hNF1 cells. Across the board, the use of a freezing container had no noticeable effect on the GPMV concentration or any of its physical properties. The use of DMSO, however, resulted in GPMVs with a higher, increased circularity and/or a decrease in eccentricity in both cell types using both mediums, with it being noticeably more effective for PB GPMVs. While the use of DMSO may not have a huge effect on the GPMV concentration preserved after cryopreservation, the GPMVs that are present appear to have a closer resemblance to those of when they were initially formed.

## Discussion

This is the first investigation of influential criteria for the production and stability of GPMVs in cell culture medium for cellular applications. Our results highlight how the addition of PFA and DTT to the cell culture medium (DMEM, 10% FBS, and 1% P/S) can produce GPMVs at the same or higher concentrations than the defined salt buffer commonly used for GPMV production. While there did not seem to be a drop in concentration between GPMVs produced from PB when resuspended in GM, changes in their circular properties hints that there could be complications with their structural integrity after resuspension in the more complex medium that is not prevalent in GPMVs produced in PM originally. Stable GPMVs in a cell culture medium could expand the potential research applications of GPMVs moving forward. Additionally, these data show that GPMVs would be more stable if they were produced by adding PFA and DTT to the suspension that will be used in later experiments rather than always producing them using PB and then resuspending them in a different medium after pelleting.

Given the lack of an assembled cytoskeleton within the GPMV’s membrane, the start of GPMV formation resembles natural cell blebbing where during the initiation step there is a localized detachment of the cell’s membrane from the cytoskeleton.^13,26^ Most commonly, this is the result from an increase in hydrostatic pressure within the cell, and the membrane begins to bulge outward and expand. Normally, the cytoskeleton reassembles and retracts back into the cell.^27^ It has been shown previously that cell exposure to sulfydryl blocking agents, like PFA, induces cell blebbing in adherent cells; however, the addition of DTT greatly increases the number of GPMVs that pinch off from the cell. The addition of DTT at low concentrations may inhibit the cortical assembly of the cytoskeleton, which is critical in the retraction of the cell bleb. While GPMVs have been shown to have cytoskeletal proteins, there is a lack of evidence to support any semblance of a fully formed cytoskeleton. With only the membrane providing structural integrity for the GPMV, changes in physical properties such as osmolarity, pH, and the concentration of specific ions could negatively affect the stability of the GPMVs formed.^28−30^ Additionally, the lack of serum in the PB may also negatively affect GPMV stability when produced from cells cultured with it.^31−33^ Our data indicate that despite the production methods implemented, approximately 10–20% of GPMVs are permeable to both 3 and 10 kDa dextran. This lack of membrane stability may pose a major hurdle to potential vehicle-based applications of these vesicles. Previous work by Skinkle et al. has attributed macromolecule permeation to shear-induced pores in the GPMV membrane.^34^

When considering ways to increase GPMV production, we first investigated the effect of higher pH as previous studies showed that increasing the pH of PB increased the number of GPMVs formed. While increasing the pH of PB resulted in a large increase in GPMVs, both in our studies and in previous studies, increasing the pH of PM did not influence GPMV generation.^35^ This would provide an explanation for why there is an increase in GPMV generation at higher pH without any noticeable changes in their physical characteristics. The mechanism for GPMV generation would be the same; however, there is an increase in bleb initiation, which in turn would increase the number of GPMVs being generated. While an increase in pH still resulted in a higher concentration of GPMVs in PM, the concentration did not jump as drastically when compared to the PB, most noticeably at pH 9.

While it has been documented that GPMVs are only produced during the first 4 h of incubation in PB, we wanted to confirm if this was the case when using PM. For both cell lines studied, we saw no noticeable increase in GPMV production after 4 h of incubation in PM and PB. Additionally, exchanging the medium with fresh PFA and DTT at the same concentration did not change the number of GPMVs produced. This indicates that the extent of GPMV production is not limited by the consumption of blebbing agents during GPMV production. The explanation for GPMV production stopping after 4 h could be due to the prolonged exposure to PFA even at such a low concentration. Higher concentrations of PFA is used to fix cells via covalent cross-linking, with lower concentrations of PFA requiring longer incubation times to fully fix cells.^36^ While, to our knowledge, it is unknown if 25 mM PFA can fix cells over this time, it could explain why GPMV production stopped after 4 h.

Adherent, human cells with different morphologies impacted the number of GPMVs generated on a per-cell basis depending on the density and time in culture. As the SCs grew to confluency, the number of GPMVs produced per cell did not show a noticeable trend as the cells grew to confluency, and it seems any per cell differences were not due to the cells growing to a higher confluency. As HEK293T cells grow to confluency, there was a noticeable decrease in the number of GPMVs generated starting at day two through day four under all conditions. However, HEK293T cells seeded at high densities (50 and 75% confluency) were dispersed more evenly throughout the dish, leaving more of the cell’s membrane exposed and increasing the number of GPMVs generated. These results highlight that the level of GPMV production increases if the cells are more evenly distributed throughout the dish. As hNF1 cells grow to confluency, they migrate away from neighboring cells and instead form multiple extensions toward neighboring cells for cell–cell interactions. Despite the cell–cell affinity being different between the two cell lines, both HEK293 and SCs do not exhibit a tight junction formation. Adherens and gap junctions differ between the two cell lines, which may influence the membrane dynamics at higher cell densities. HEK cells express a combination of N- and E-cadherins to form more tightly bound monolayers in vitro. However, GPMV production is most likely a result of differences in membrane stability because of cytoskeletal and intermediate filament differences. HEK cells exhibit a stochastic microtubule organization and a distinct cortical actin structure,^37^ while SCs possess highly aligned stress fibers and a high degree of actin polymerization in leading lamellipodia.^38^ This distinction in the cytoskeletal arrangement was linked to acidic calponin, which regulates actin-myosin interactions. The overexpression of acidic calponin in HEK 293 cells induces a neuron-like morphology characterized by long processes and reorganization of microtubules.^39^ GMPVs are devoid of F-actin and intermediate filament proteins, despite the importance of actin polymerization and myosin-actin contraction for bleb retraction. Alpha-tubulin is present in GPMVs and may provide stability.^40^ The more pronounced cortical actin structure in HEK cells may increase the retraction of forming blebs compared with SCs, resulting in higher GPMV yields for SC cultures.

Cryogenic storage of GPMVs showed that while there is a loss in GPMV concentration, more than half of the GPMVs are stable following cryogenic freezing and thawing. When only considering the concentration of GPMVs post cryogenic storage, the use of 10% DMSO and an IPA freezing container did not appear to have a noticeable effect on increasing the GPMV concentration significantly. The use of DMSO induced an increase in the average diameter and circularity, as well as a decrease in the average eccentricity, when compared to the samples that did not use DMSO. In contrast, the rate of freezing did not make a significant impact on the stability of GPMVs. This suggests that ice crystal formation influences the stability of larger GPVMs and seems to cause deformations in the GPMVs imaged. When GPMVs were frozen in PB, the use of DMSO had a very significant impact on the physical profile of GPMVs analyzed, with the average diameter and circularity increasing and average eccentricity decreasing in both cell types at both storage temperatures. This may be due to the GPMV buffer having a simpler salt composition and lacking serum that may influence how the crystalline network formed during freezing.^41^ While DMSO is toxic to cells outside of cryogenic storage, its inclusion seems to have a positive impact on preserving GPMV structure in cryogenic storage, particularly for larger GPMVs. This type of cold storage may be required for on-demand use or transportation.

## Conclusions

Here, we tested the potential of producing GPMVs using PFA and DTT in a medium suitable for cell culture and compared it to the salt buffer historically used for GPMV production. We found that GPMVs were readily produced in PM, producing GPMVs at an equal or higher concentration than PB. The majority of GPMVs are generated within 4 h of incubation, and there was no significant increase in GPMV concentration with longer incubation durations. GPMV concentrations were increased when cells were blebbed at a higher pH and when they were seeded at a higher confluency and dispersed more around the vessel, particularly for HEK293T cells. These findings illustrate how GPMV production can vary based on the cell type and morphology, cell culture conditions before and during production, and what medium is used for GPMV production. Production of GPMVs using cell culture media shows how GPMV formation is largely dependent on PFA and DTT and not with the use of serum. While GPMVs remain primarily as a model for studying the plasma membrane, a clear understanding of production, storage, and stability may help facilitate the development of new applications for GPMVs in the future.

## Supporting Information Available

Raw images of collected GPMVs from HEK293T and hNF1 cells in different media formulations incubated with fluorescent dextrans to illustrate passive permeability (PDF)
