# Supplementary material for: Cell Line and Media Composition Influence the Production of Giant Plasma Membrane Vesicles
Source: ACS Biomater Sci Eng. 2024 Feb 20;10(3):1880–91. doi: 10.1021/acsbiomaterials.3c01596 (PMC10934252; doi:10.1021/acsbiomaterials.3c01596)
Supplement: Supplementary file 1 — ab3c01596_si_001.pdf [file ab3c01596_si_001.pdf]

Cell line and Media Composition Influence the Production of Giant Plasma Membrane Vesicles

*William Doherty[1], Sarah Benson[1], Lisa Pepdjonovic[1], Abigail N. Koppes[1,2,3], Ryan A. Koppes[1]**

1. Department of Chemical Engineering
   Northeastern University
   360 Huntington Ave.
   Boston, MA 02115, USA
2. Department of Biology
   Northeastern University
   360 Huntington Ave.
   Boston, MA 02115, USA
3. Department of Bioengineering
   Northeastern University
   360 Huntington Ave.
   Boston, MA 02115, USA

*Corresponding Author

Ryan A. Koppes
360 Huntington Ave
339 Mugar
Boston, MA 02115, USA
r.koppes@northeastern.edu

Supplemental Figures

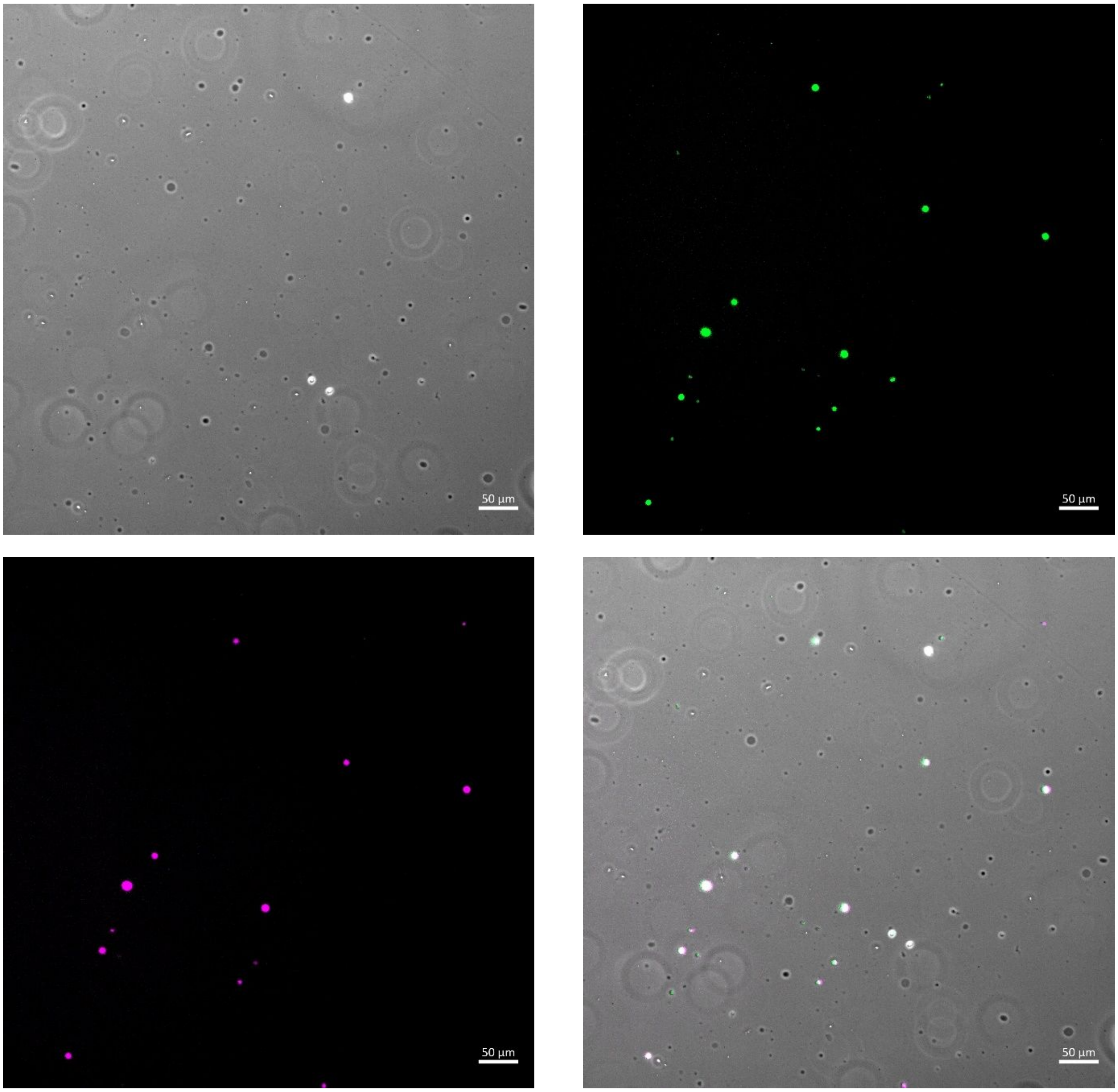

Figure S1 GPMVs produced from HEK293T cells in PM after suspennsion in Dextran mixture followed by pelleting and resuspennsion in Media: a) Brightfield b) Fluorescein c) Alexa Fluor 647 d) Merged.

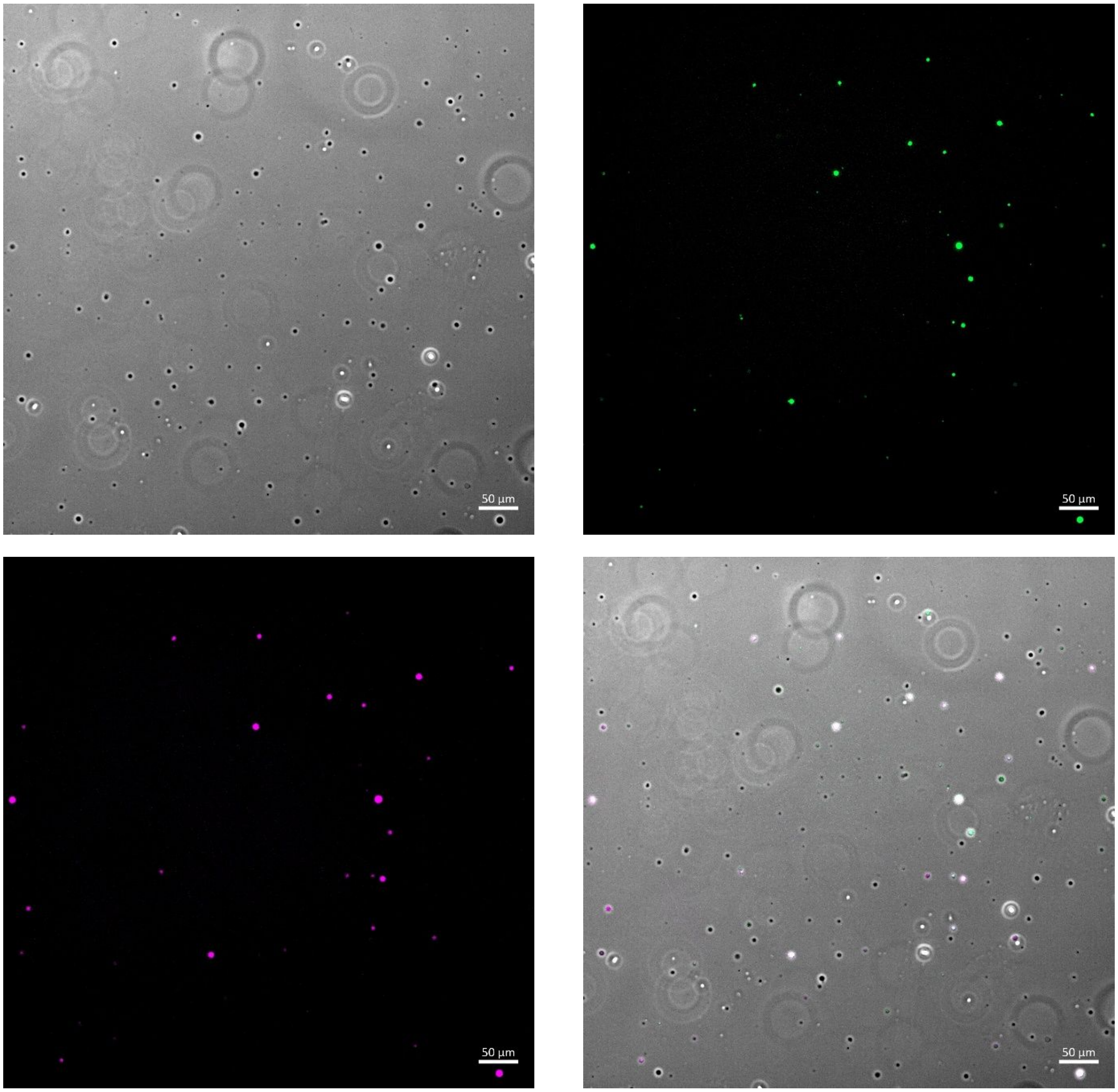

Figure S2 GPMVs produced from HEK293T cells in PB after suspennsion in Dextran mixture followed by pelleting and resuspennsion in Media: a) Brightfield b) Fluorescein c) Alexa Fluor 647 d) Merged.

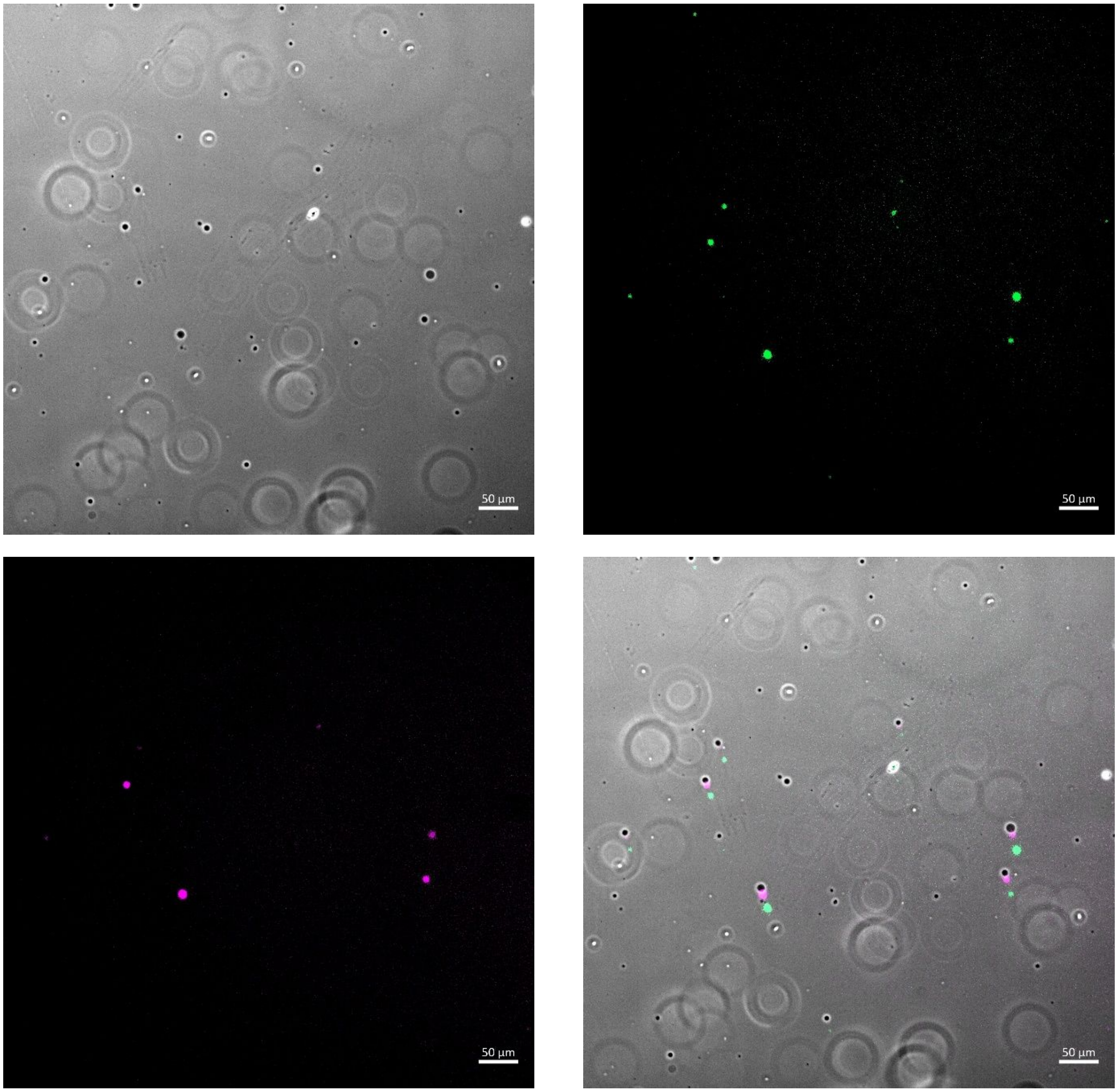

Figure S3 GPMVs produced from hNF1 cells in PM after suspennsion in Dextran mixture followed by pelleting and resuspennsion in Media: a) Brightfield b) Fluorescein c) Alexa Fluor 647 d) Merged.

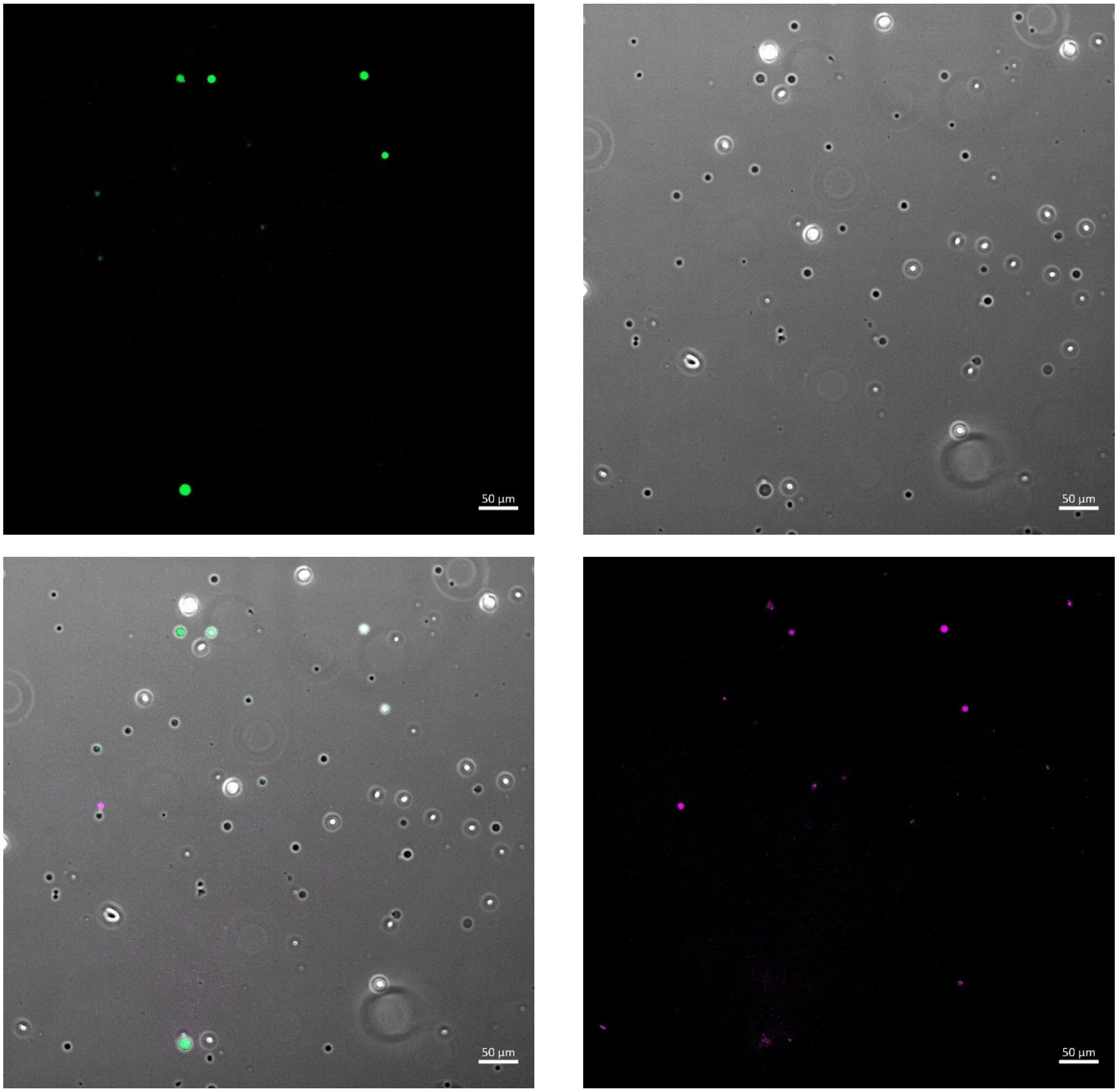

Figure S4 GPMVs produced from hNF1 cells in PB after suspennsion in Dextran mixture followed by pelleting and resuspennsion in Media: a) Brightfield b) Fluorescein c) Alexa Fluor 647 d) Merged.
